# Challenges and Opportunities for Consistent Classification of Human B Cell and Plasma Cell Populations

**DOI:** 10.3389/fimmu.2019.02458

**Published:** 2019-10-18

**Authors:** Ignacio Sanz, Chungwen Wei, Scott A. Jenks, Kevin S. Cashman, Christopher Tipton, Matthew C. Woodruff, Jennifer Hom, F. Eun-Hyung Lee

**Affiliations:** ^1^Lowance Center for Human Immunology, Emory University, Atlanta, GA, United States; ^2^Division of Rheumatology, Department of Medicine, Emory University, Atlanta, GA, United States; ^3^Division of Pulmonary, Allergy, and Critical Care, Department of Medicine, Emory University, Atlanta, GA, United States

**Keywords:** B cells, naïve, memory, transitional, ABC, DN2, atypical B cells, Breg

## Abstract

The increasingly recognized role of different types of B cells and plasma cells in protective and pathogenic immune responses combined with technological advances have generated a plethora of information regarding the heterogeneity of this human immune compartment. Unfortunately, the lack of a consistent classification of human B cells also creates significant imprecision on the adjudication of different phenotypes to well-defined populations. Additional confusion in the field stems from: the use of non-discriminatory, overlapping markers to define some populations, the extrapolation of mouse concepts to humans, and the assignation of functional significance to populations often defined by insufficient surface markers. In this review, we shall discuss the current understanding of human B cell heterogeneity and define major parental populations and associated subsets while discussing their functional significance. We shall also identify current challenges and opportunities. It stands to reason that a unified approach will not only permit comparison of separate studies but also improve our ability to define deviations from normative values and to create a clean framework for the identification, functional significance, and disease association with new populations.

## Introduction

B cells constitute a critical arm of the immune system and are responsible for the short-term and long-term generation of humoral antibody responses. B cells also carry out antibody-independent functions including: antigen-presentation, modulation of T cell differentiation and survival, and production of both regulatory and pro-inflammatory cytokines (1–4). Finally, B cells play a critical role in the formation of secondary and tertiary lymphoid tissue. A growing number of publications reflect their indispensable role in the generation of protective and pathogenic antibodies and their functional versatility, as well as their potential utility as disease biomarkers and therapeutic targets. Their behavior and value as biomarkers during changes in disease activity, whether spontaneous or in response to treatment, has also been an area of strong exploration. Unfortunately, there is growing difficulty in reconciling different and often contradictory studies. At the core of this problem are: the use of limited and inconsistent phenotypic markers, the use of pauci-color flow cytometry, inappropriate and imprecise extrapolation of the significance of individual markers, and the forced inclusion of multiple B cell subsets within larger and heterogeneous populations. These problems are compounded by the assignment of functional properties (regulation, activation, anergy) and developmental connotations on the basis of surface phenotype rather than precise functional characterization and/or molecular markers (transcriptional factors/networks).

## Peripheral B Cell Diversity: Emphasis on Human Phenotypes

**Figure 1:**
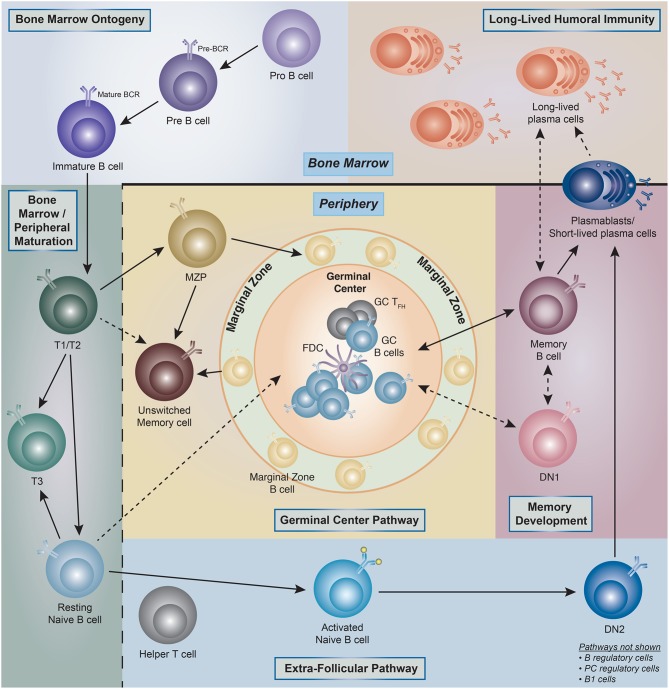
Human B cell ontogeny. Illustration of the current understanding of B cell ontogeny in human B cells from late bone marrow (pro- and pre-B cells) through peripheral activation pathways, and into antibody secreting cells (ASC)/long-lived plasma cells compartments (PC). (→) Denotes clear literature supported associations, whereas (- - - >) represent associations which are theoretical but have literature supported plausibility. T1, transitional type 1 B cells; T2, transitional type 2 B cells; T3, transitional type 3 B cells; MZP, marginal zone precursor B cells; DN1, double-negative (IgD– CD27–)−1 B cells; DN2, double-negative (IgD– CD27–)−2 B cells; GC, germinal center; FDC, follicular dendritic cell.

Widely accepted schemes of human B cell development are modeled on knowledge derived from mouse studies, and indeed, fractions equivalent to murine pro-B, pre-B, immature, transitional B, and naïve B cells have been identified in human bone marrow (5–7). Current knowledge of peripheral human B cells stems mostly from tonsil analyses and a plethora of peripheral blood studies complemented by more limited analyses of spleen cells and non-malignant lymph nodes (8–29). The combination of those studies has identified the human counterpart to transitional and mature B cell compartments with the latter containing naïve B cells; germinal center (GC) B cells; memory B cells; and antibody-secreting cells (ASC) (25). Of note, with the possible exception of GC cells, all human B cell populations found in lymphoid tissues can also be demonstrated in the peripheral blood lymphocytes (PBL) including the ambiguously coined tissue-based memory cells further discussed below. These populations can be considered as parental populations comprised of different B cell subsets as it will be discussed later in the review (Figure 1).

From a developmental standpoint, mouse B cells are generally classified into B1 and B2 (follicular) B cells and marginal zone (MZ) B cells, with B1 and MZ B cells sharing important functional properties and in particular, the ability to generate a fast and intense burst of ASC against particulate bacterial antigens (30). In addition, mouse studies have also defined two major pathways of B2 cell activation and differentiation into ASC: (1) the GC pathway leading to the generation of long-lived memory cells and plasma cells; and (2) the extra-follicular pathway responsible for the generation of B cell blasts and short-lived plasmablasts (PB) (31). Of note, the extrafollicular pathway can also generate long-term memory and long-lived PC in a T cell independent fashion (32–34). While MZ B cells have been well-described in the human spleen and a circulating MZ equivalent is also recognized in the peripheral blood, the existence, significance and phenotypic identifiers of human B1 cells remain in dispute. Indeed, while an original report identified a population of human B cells sharing phenotypic (CD20+, CD27+, CD43+, CD70–), B cell repertoire, and functional features of the murine B1 population, subsequent analysis by the same group and others shed some doubt on the quantitation of these cells (35–39).

## Phenotypic Markers of Human Peripheral B Cells. Basic Experimental and Analytical Approach

**Figure 2:**
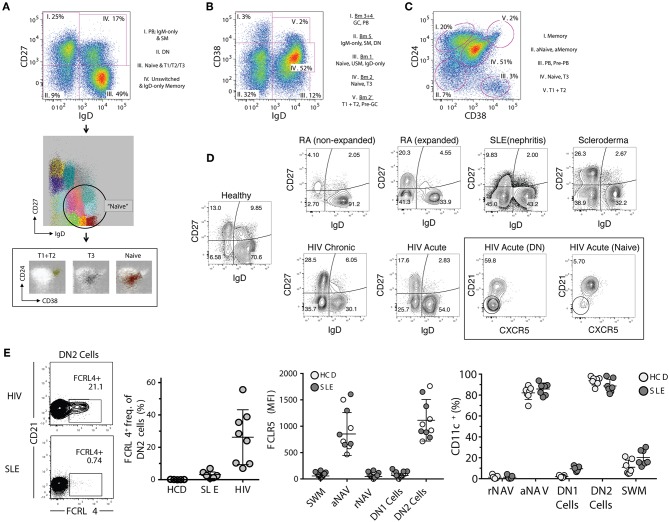
Proposed B cell gating strategies. **(A)** CD27 vs. IgD gating strategy of CD19+ cells in a normal healthy donor representing four distinct B cell fractions: (I) switched memory (SM) B cells with associated plasmablasts (PB), (II) CD27– IgD– DN (double-negative) B cells, (III) global naïve B cell gate which includes transitional types 1–3 populations (associated FLOCK plots), (IV) Unswitched memory/IgD-only memory B cells. **(B)** Bm1–Bm5 gating strategy which identifies five B cell fractions which have more difficulty resolving memory from effector B cells, and naïve B cells from memory. **(C)** CD24 vs. CD38 gating strategy which defines five B cell fractions with good separation of memory from naïve (excluding the fraction II which represents combined activated memory and activate naïve) and naïve from transitional B cell populations. Better population resolution can be attained by subgating from the naïve population within the CD27 vs. IgD strategy followed by this CD24 vs. CD38 strategy. Of note is that transitional type-3 (T3) B cells cannot be resolved in any strategy without the use of MitoTracker® Green (MTG). **(D)** Representative FACS files showing (CD27– IgD–) DN B cells in a population of rheumatoid arthritis (RA), systemic lupus erythematous (SLE), scleroderma, and both acute and chronic human immunodeficiency virus (HIV) infected patients. Additionally, the DN population can be further defined by markers such as CXCR5. **(E)** Derived from Jenks et al. (41), Immunity, showing FcRL4 expression in atypical memory B cells from HIV patients but not SLE DN B cells, and the reciprocal expression pattern for FcRL5 (high in SLE but not HIV). This heterogeneity in expression patterns is indicative of multiple DN B cell populations, and can only be resolved by further marker subgating on DN B cells. Additionally, CD11c is highly expressed in SLE B cells and is indicative of DN2 B cells (activated effector cells) and are very different from the proposed atypical memory B cells of HIV which are thought to be anergic. PB, plasmablasts; DN, CD27– IgD– double negative B cells; T1, transitional type 1 B cells; T2, transitional type 2 B cells; T3, transitional type 3 B cells; GC, germinal center-like B cells; SM, switched memory B cells; USM, unswitched memory B cells; aNaive/aNAV, activated naïve B cells; Naïve/rNAV, resting naïve B cells; DN1, double negative 1 B cells; DN2, double negative 2 B cells, HIV, human immunodeficiency virus; SLE, systemic lupus erythematosus.

All major parental populations of human peripheral B cells can be identified using a relatively small number of surface phenotypic markers including CD19, CD20, IgD, CD27, CD38, and CD24 (25). With the exception of plasma cells, the expression of CD19 and CD20 follows a largely overlapping pattern and their concomitant measurement may be redundant. Generally, these markers are used in conjunction with non-B cell lineage markers (exclusion channel), for positive identification of members of the B cell lineage and exclusion of non-B cells from the analysis. When only a limited number of markers are available, we prefer to use CD19 since the intensity of expression of this marker may provide valuable information regarding B cell activation, and expression may help differentiate between short-lived and long-lived PC, as will be discussed in further detail below (40). In turn, while the absence of CD20 is a valuable indicator of ASC in peripheral B cell analysis, enumeration of all ASC can also be achieved through the combination of CD27 and CD38 with the CD27hiCD38hi fraction containing both CD20– ASC as well as a small fraction of CD20+ ASC (see below). In all, we believe that proper analysis of the major canonical human B cell subsets can be achieved through, and requires, the analysis of 7-markers combined with proper exclusion of dead cells and cellular doublets. The recommended markers include: (1) non-B cells exclusion markers such as CD3 and CD14; (2) CD19; (3) IgD; (4) CD27; (5) CD38, (6) CD24; and (7) CD21. This combination of markers enables the analysis of human B cells through a combination of the two more widely used classification schemes: IgD vs. CD27; and IgD vs. CD38 (Figures 2A,B). The latter approach classifies B cells using the Bm1–Bm5 nomenclature originally derived from the study of mature B cells (Bm) in the human tonsil (8). Of note, this nomenclature was designed for the classification of B cells in human lymphoid tissue with the help of additional markers including CD10, CD44, CD77, and CD23 (8). When applied to the peripheral blood on the basis of IgD and CD38 expression, this approach is less categorical than the IgD/CD27 approach: as it fails to separate transitional cells (IgD+ CD38hi) from pre-GC cells (Bm2'), coalesces different types of memory B cells (10), does not separate resting naïve cells (Bm1) from IgD+ unswitched memory cells (10), and does not distinguish between conventional CD27+ memory cells and IgD/CD27 double negative cells, which in turn contains a heterogeneous population of cells including atypical/tissue-based/exhausted memory cells and activated extrafollicular PB precursors (25, 41–46). Accordingly, we recommend to base the initial categorization of parental B cell populations on the combination of IgD, CD27, and CD38 together with CD24 (Figures 2A,C). In addition, we strongly recommend the inclusion of CD21 in the core marker set as its expression enables the recognition of activated cells within all parental B cell populations and possibly, as further discussed below, of developmentally distinct B cells (Table 1). While largely overlapping with CD19, the inclusion of CD20 offers additional discriminating power to identify ASC.

**Table 1:** Classification of blood human B cells using seven core markers.

**B cell population (CD19+** **unless otherwise noted)**		**Core markers**	**Additional markers**	**Function/properties**
Transitional	T1/T2	IgD+ CD27– CD38++ CD24++	CD10++/+ IgM++ MTG+ CD10/IgM expression= T1 > T2 > T3	Developmental precursor
	T2-MZP	IgD+ CD27– CD38 ++ CD24++ CD21++	CD10+ IgM++	MZ precursor
	T3	IgD+ CD27– CD38+ CD24+ CD21+	CD10+/– IgM+ MTG+	Developmental precursor or activated naïve
Naïve	Resting	IgD+ CD27– CD38+ CD24+ CD21+	IgM+ MTG–	Antigen inexperienced mature cells
	Activated	IgD+ CD27– CD38– CD24– CD21–	IgM+ MTG+ CD95+ CD23– CD11c+ T-bet+ FcRL5+ SLAMF7+ CXCR5–	Precursor of short-lived PB and GC reactions
	Anergic	IgD+ CD27– CD38+/lo CD24+ CD21–	IgMlow/–	Hypo-responsive. Maintenance of tolerance
Memory	Unswitched	IgDlo CD27+ CD38+/lo CD24+ CD21+	CD1c+ IgM++	Natural memory. MZ equivalent
	Pre-switched	IgD– CD27+ CD38+/lo CD24+ CD21+	IgM+	Pre-switch memory; early IgM memory IgG memory precursor
	Switched resting	IgD– CD27+ CD38+/lo CD24+ CD21+	IgG/IgA+ CD95–	Pre-existing memory reservoir
	Switched activated	IgD– CD27+ CD38– CD24– CD21–	IgG/IgA+ CD95+ CD86+	Effector memory-PB/PC precursor
	Atypical tissue-based	IgD– CD27– CD38lo CD24lo CD21–	FcRL4+ IgM/IgG/IgA+ FcRL5+	Mucosal surveillance; exhausted memory; BCR hypo-responsive memory
Double negative (DN)	DN1	IgD– CD27– CD38+ CD24+ CD21+	FcRL4– IgM/IgG/IgA+ FcRL5– CXCR5+	Memory precursors
	DN2	IgD– CD27– CD38– CD24– CD21–	IgM/IgG/IgA+ T-bet+ CD11c+ FcRL5+ CXCR5– SLAMF7+	Extrafollicular ASC precursors
		IgD– CD27– CD38– CD24– CD21–	FcRL4+	Atypical/Tissue-based memory
Antibody secreting cells	Early PB	IgD– CD27lo/+ CD38++ CD24–	CD20+/– HLA-DR+ CD138–	Naïve and memory-derived PB precursors
	PB	IgD– CD27 ++ CD38+++ CD24–	CD20– HLA–DR+ CD138– Ki67+	Antibody secretion
	PC	IgD– CD27 ++ CD38+++ CD24–	CD19+/– CD20– CD138+	Antibody secretion
Regulatory B cells (Breg)		Multiple surface phenotypes corresponding to different core populations and subsets –Pro-B10 and B10 (no unique markers; defined by IL-10 production) –IL-35-producing Breg (defined by IL-35 production)		Down-regulation of T cell and monocyte inflammatory and autoimmune responses
Regulatory plasma cells (PCreg)		IgD– CD27++ CD38++	CD19+/– CD138+ IL−10+ or IL−35+ IgMhi or IgA+ (separate populations)	Suppress immune responses including anti-tumor responses
B1 cells		IgD++ CD27+	IgM+ CD43+ CD70– CD11c+ CD14+ CD5+/–	Production of natural autoantibodies

***Core Markers**: CD19, IgM, IgD, CD27, CD38, CD24, CD21. **Additional Markers**: IgM, IgG, IgA, CD20, CD11c, FcRL5, FcRL4, CD138, CD95, CD80/86, CD23, T-bet, Ki-67, others*.

As further discussed elsewhere in this review, the use of additional markers may significantly increase the investigators' ability to discriminate finer subsets of different functional properties and developmental origin (Table 1). Valuable makers whose use and discriminatory power has been well-documented in multiple studies include: IgM, IgG, IgA, CD10, CD23, CD80, CD86, CD69, CD95, CD11c, CD22, CXCR5, FcRL4, and FcRL5. In addition, the use of CD138 identifies more mature PC. A smaller number of studies have used CD25 as a marker of activation and in at least one study as a marker of B regulatory cells (47, 48). In addition, CD71 is a valuable marker of early memory B cell activation (49). Finally, CD5 and CD43 have been used to identify B1 cells. The value of CD5 for this purpose however, has been negated by multiple studies as this marker can be expressed by multiple B cell populations at least in part as a result of B cell activation (9, 50, 51).

In addition to surface markers, the classification of B cells can be powerfully aided by measuring the expression of proliferative markers (e.g., Ki-67); transcription factors (41, 52–60) and intracellular or secreted cytokines (1, 3). However, a comprehensive discussion of these valuable markers is outside the scope of this review. Accordingly, we will only address the significance of the expression of the transcription factor T-bet, given the prominence it has gained in the B cell literature over the last few years (61).

## Classification of Canonical B Cell Populations in the Human Peripheral Blood

As previously mentioned, a limited number of surface markers suffice for a consistent and quantitative measurement of all major B cell populations (Table 1). The canonical B cell populations thus recognized in the human peripheral blood are discussed in detail below and include: transitional cells, naïve B cells, memory B cells, circulating marginal zone (MZ) B cells, atypical memory B cells, and ASC. A more precise characterization of these canonical populations and of the distinct subsets they contain requires the use of additional markers (Table 1).

### Transitional B Cells (T; Canonical Phenotype: CD19+, IgDlo/+, CD27–, CD24++, CD38++)

In the mouse, newly formed bone marrow immature B cells differentiate through sequential transitional stages of maturation into functionally competent follicular naïve B cells, a process originally thought to be limited to the spleen, but that it is now known to also take place in the bone marrow (62, 63). The transitional B cell differentiation process is replicated in humans with T1, T2, and T3 circulating transitional fractions recognized in the peripheral blood (9, 22, 63). These consecutive fractions differ in their relative level of expression of CD24, CD38, CD10, and CD9 as well as IgM, all of which are gradually downregulated from T1 → T3 (Figures 2A,C) (9, 22). Transitional cells share with memory cells and activated naïve cells, the ability to retain mitochondrial dyes such as rhodamine or MitoTracker® Green owing to the absence of the ATP-binding cassette (ABC) B1 transporter (64). Similar to mice, the human spleen also contains a MZ precursor (MZP) representing a branching point of T2 cells characterized by high levels of CD21 (13, 22), a feature shared by a population of bone marrow transitional cells (65). Similar cells can be detected in the PBL on the basis of their expression of a CD45RB glycoform recognized by the MEM55 antibody (21, 66). In contrast to other transitional cells, a fraction of MZP express the (ABC)B1 transporter and accordingly, do not retain mitochondrial dyes.

The actual significance of T3 cells remains unknown as, in the mouse, these cells have been proposed to represent either anergic B cells (67), or the immediate precursor of mature naïve B cells (68); a characterization disputed by others (69). In turn, human cells with a T3 phenotype may represent either late transitional naïve precursors in the context of post-rituximab B cell repopulation (22) or early activated naïve B cells in active SLE (70).

### Naïve B Cells (N: Canonical Phenotype: CD19+, IgD+, CD27–, CD38+/–, CD24+/–)

Human naïve B cells are typically defined by the expression of high levels of IgD and positive IgM staining, although at lower levels than in transitional cells. Using the core flow panel, N cells can be separated from transitional cells by their down-regulation of CD38 and CD24 which in N cells are both expressed as a continuum ranging from low-to-negative expression; and lack of CD27 expression (Figure 2A). Extended panels can further separate N cells based on their lack of expression of CD10 and lack of retention of mitochondrial dyes. Contrary to transitional cells, characterized by higher expression of IgM in a narrower MFI range, N cells express a wider continuum of surface IgM levels that include fractions with low-to-negative levels. The latter smaller fraction represents <2% of blood B cells and is characterized by the absence of IgM (IgD+, IgM– naïve, or BND), representing anergic autoreactive cells, a phenotype that has been extended to a larger fraction of IgMlow IgD+ cells encompassing 30% of all N cells (71). The larger low expression fraction is consistent with the abundance of autoreactive anergic B cells initially demonstrated in transgenic mice and then in wild-type mice (67, 72). It should be noted however, that IgM downregulation can also be induced by BCR activation and that expansions of IgMlow N cells with activated phenotype are observed in autoimmune diseases such as SLE (71). These observations should therefore, temper the use of IgM levels in isolation of other markers to define anergy or activation in the context of immune stimulation.

The use of additional markers recommended for the extended panels, such as CD21 and CD23 as well as traditional markers of activation such as CD80, CD86, CD95, and CD25, clearly discriminates distinct naïve B cell subsets. Thus, the characteristic phenotype of blood (resting) naïve B cells includes constitutive expression of CD21 and CD23 established during maturation and the absence of CD80, CD86, CD95, or CD25. The latter set of markers is only expressed by a minor fraction of N cells (<5%), in unperturbed healthy controls and gets differentially modulated upon B cell activation either through the BCR, CD40L, or TLR engagement in the context of IL-4 and other cytokines (73–79). It is important to recognize that significant differences exist in the intensity and kinetics of upregulation of these markers in response to different stimuli (80–83). Accordingly, it is recommended that the same marker and protocol be used when comparative analysis of naïve B cell activation is undertaken.

**Figure 3:**
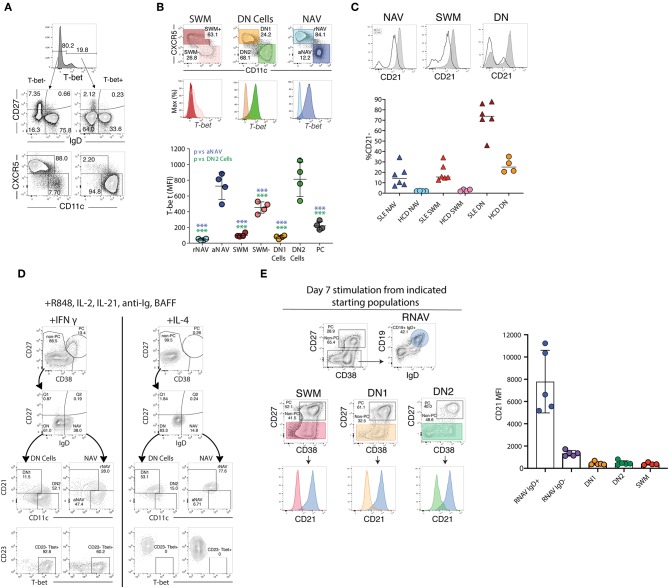
T-bet, CD21, and CD11c expression *ex vivo* and after stimulation. **(A)** The majority of T-bet high B cells are IgD–CD27– DN or IgD+CD27– naïve B cells with a CD11c bright and CXCR5– phenotype characteristic of DN2 and activated naïve B cells, staining of CD19 B cells from a representative SLE patient. **(B)** Activated naïve and DN2 have the highest levels of intracellular T-bet staining. Gating and histograms are shown for a representative SLE patient is shown on top and quantification of T-bet mean fluorescence intensity for four SLE patients is shown below. Note, while CXCR5– SWM and CD27++ CD38++ PC express some T-bet their MFI is still significantly lower than that of DN2 and activated naïve. **(C)** Stimulation of HCD naïve B cells with TLR7 agonist R848, cytokines, and interferon gamma but not IL4 results in both plasma cell differentiation and increased T-bet and CD11c expression with concomitant loss of CD21 and CD23 expression. **(D)** Naive B cells from both HCD and SLE patients gain CD11c and lose CD21 in response to stimulation with interferon gamma, R848, and cytokines. **(E)** CD21 expression from flow sorted *in vitro* differentiated B cell populations (starting population indicated above center flow plots). There was a reduction of CD21 expression within all cultures (as compared to residual CD19+ IgD+ undifferentiated resting naïve B cells), independent of starting B cell population, suggesting that the loss of CD21 is indicative of a B cell activation state and recapitulates the *in vivo* phenotype of DN2 and activated naïve B cells. **(B,D)** were adapted from Jenks et al. (41).

Valuable information can also be obtained through the expression of CD23 and CD21, two markers commonly used in the study of human B cells and whose expression is modulated during both B cell development and activation (65). CD21 and CD23, which in mouse and human spleen are fundamental for the adjudication of follicular and marginal zone phenotypes (13, 24, 84), are expressed universally by unperturbed human blood naïve B cells and their downregulation is observed in a major fraction of naïve B cells in several immunological conditions, including SLE (41, 85). Despite these observations, stemming from murine models and extrapolations made from the analysis of tonsil B cells and diverse *in vitro* stimulation conditions, the expression or lack thereof, of CD23 in human N cells has been interpreted in opposite ways. Thus, it was initially reported that tonsil naïve B cells upregulate CD23 during their differentiation (CD23– Bm1 to CD23+ Bm2) to GC centroblasts (Bm3). Yet, the same and subsequent studies also demonstrated the absence of CD23 in GC cells and in an activated (CD71+) intermediate population postulated to represent the early stages of naïve differentiation into GC cells or GC founders (86, 87). Consistent with an activated phenotype of CD23– N cells, multiple studies have identified expansions of CD23– B cell populations in SLE (40, 41, 71, 88, 89). These studies include recent detailed functional, transcriptional, and epigenetic characterization of activated naïve B cells marked by over-expression of T-bet, CD11c, SLAMF7, FcRL5 and other activation markers including CD80/CD86 and CD69, as well as downregulation of CD21 and CD23 (Figures 2C, 3A,B) (40, 41, 90). However, expansion of activated naïve B cells in SLE has also been postulated on the basis of CD23 upregulation (74). This work however also described an expansion of CD23-negative naïve cells that were attributed to possible contamination with memory cells. Unfortunately, the absence of IgD, CD27, and CD23 co-staining precluded a conclusive identification of the relevant populations and even larger proportions of CD27– CD23– cells expressed CD80 and CD86 in SLE relative to CD23+ cells in healthy controls. Of interest, the recently described DN2 population (IgD– CD27– CD23– CD11c+ Tbet+), which is highly expanded in active SLE and which represents the progeny of activated extrafollicular naïve cells, could have accounted for the expansion of CD23– cells (42).

On the basis of what is known about the stimuli that upregulate CD23 expression on human B cells, it is certainly possible that activated naïve B cells could express different phenotypes in different conditions depending on a number of variables such as the duration of stimulation, type of T cell help, and cytokine milieu (75, 89, 91–93). Thus, IL-4 seems to be the main inducer of this marker after either BCR or CD40 stimulation and this induction is inhibited by IFNγ (77, 91, 92) (Figure 3). Notably, IL-4 and IFNγ exert a similarly reciprocal regulation on the differentiation of T-bet+ B cells induced in SLE (41, 94–103).

Similar to CD23, as further discussed below, CD21 downregulation identifies expanded fractions in several diseases characterized by increased B cell autoreactivity including SLE, CVID, Sjogren's syndrome, and RA (12, 41, 85, 104–107). CD21 downregulation also marks activated memory cells whether in normal vaccination responses, HIV and malaria infection, or in memory cells expanded in response to checkpoint inhibitors. This feature is shared by other activated B cells including: CD11c+ activated naïve and DN2 cells, atypical and tissue-based memory cells, and T-bet+ B cells (15, 95, 108–111). Nevertheless, despite the preponderance of evidence for the activated phenotype of CD21low B cells within the naïve compartment and in particular in autoimmune diseases such as SLE, CD27– CD21low B cells have also been characterized as anergic B cells in a fraction of patients with RA and CVID (112). As previously discussed however for CD23, these studies focused on cells defined as CD27– CD21low without consideration of IgD expression and accordingly, they would have also included isotype-switched CD27– cells with hypo-responsiveness to anti-IgM stimulation (41, 93). One additional caveat that must be made in regards to CD21low B cells, is that although low in frequency, early transitional B cell populations (e.g., T1 B cells) may also be CD21low which can be isolated utilizing CD38, CD24, and/or CD10 (65).

### Memory B Cells (M; Canonical Phenotype: CD19+, CD27+, CD38+/–, CD24+/–)

There is fairly universal consensus that human memory cells can be defined through the expression of CD19 and CD27 although CD19+ CD27+ blood cells also include antibody-secreting PB. In turn, M B cells and PB can be effectively differentiated with the core flow panel on the basis of CD38 expression. Thus, while M cells express intermediate to low levels of CD38 (Bm5 and Bm5' subsets, respectively, under the Bm classification), PB are CD38hi at levels that readily separate from memory cells. Also useful for this differentiation is the lack of expression of CD20 on most PB as further discussed below.

The assignation of a memory phenotype to circulating CD27+ B cells is based on the observation that, like germinal center cells which initiate CD27 upregulation (24, 113, 114), these cells accumulate significant rates of somatic hypermutation (14, 16, 17, 113, 115). Moreover, CD27+ B cells represent the main source of recall responses (49, 109, 116, 117). Finally, CD27+ B cells are endowed with enhanced differentiation into ASC and a distinct transcriptional program (118, 119).

Several memory subsets have been reported that can be identified with core markers (43, 120, 121). The first critical distinction separates IgD+ CD27+ and IgD– CD27+ cells. The IgD+ subset contains both IgM+ (CD27+ IgDlo IgM++; unswitched memory cells), and IgM-negative cells (IgD–only switched memory cells). Whereas, IgD– CD27+ cells comprise both IgM-only (pre-switch memory cells) and IgG, IgA, or IgE expressing cells (switched memory) (16, 17, 122–124). The unswitched nature of the IgD+ IgM+ populations and their separate origin relative to pre-switch memory, is consistent with the generation of the former subset in CD40L-deficient hyper-IgM patients unable to receive T cell help or form germinal centers and undergo class switch (although the potential for class-switching remains under normal conditions) (125). In turn, a pre-switched state capable of undergoing subsequent class switch has been suggested for IgM-only CD27+ memory cells (18, 126). It seems likely that IgM-only pre-switch cells represent the human counterpart of murine IgM memory cells generated in the early phases of the GC reaction that serve as a substrate for affinity matured isotype-switched cells during subsequent rounds of GC reactions (18, 117, 127). In contrast, despite lack of universal consensus (121), there is substantial evidence to indicate that IgD+ IgM+ unswitched memory cells may represent circulating marginal zone B cells (13, 84). Other studies however have proposed a GC origin for these cells on the basis of their mutational Bcl6 pattern (117). These observations taken together, lead us to propose that when IgM is available within an extended panel, memory B cell populations be further separated on the basis of both IgM and IgD expression as they represent distinct memory pools. Finally, IgD+ IgM–, CD27+ switched memory cells constitute a unique compartment representing a small but distinct fraction whose biology needs further clarification. Interestingly, IgD-only memory cells are characterized by an unusually large load of somatic hypermutation and may represent the origin of, heavily mutated IgD+ multiple myeloma (also uncommon) (128). While these cells appear to be enriched for autoreactivity (129, 130), their role in physiological and pathological conditions remains to be fully elucidated. Recent work however suggests that they may mediate important antimicrobial responses in the respiratory mucosa by arming basophils with IgD antibodies. In turn, faulty regulation of this mechanism could lead to the development of auto-inflammatory syndromes (131).

As for naïve B cells, resting and activated subpopulations can be identified through the use of markers included in the core and ancillary panels. Thus, constitutive expression of CD21 is maintained in resting memory cells and lost upon activation as illustrated by substantial increases of CD21lo CD27+ memory cells in HIV infection (108, 132, 133), influenza recall responses (106), and in patients with SLE and RA (110, 121, 134–136). Activated memory cells also upregulate CD95, CD80, and CD86 but as previously indicated, the correlation between these markers with CD21 downregulation is incomplete and accordingly, the same set of markers should be employed to establish changes in memory cell activation between samples (80, 110, 134, 137). Finally, upregulation of CD71 appears to be a helpful marker of early activation in proliferative antigen-specific memory cells and new germinal center products that differentiate into antibody-secreting PB (49, 106).

### Atypical and Tissue-Based Memory B Cells (Canonical Phenotype: Double Negative, DN: CD19+, IgD–, CD27–, CD38+/–, CD24+/–)

SLE is characterized by large departures from normal immunological homeostasis that are commonly reflected in the peripheral blood. This behavior has facilitated the identification of distinct populations that under normal circumstances are under-represented in the blood, including a canonical subset characterized by the absence of both naïve (IgD) and conventional memory markers (CD27), termed double negative B cells (DN). Typically representing <10% of all PBL CD19+ cells, DN cells can contribute in excess of 40% of all B cells in active SLE and may become the largest circulating population of isotype switched IgD– cells (23, 41, 136) (Figures 2A,D). Distinguished by a CD19high activated phenotype, SLE DN cell expansion has been associated with: active disease, African-American descent, adverse clinical outcomes, and poor response to B cell depletion therapy (23, 41, 135). More recent work discussed below, has established their identify as activated effector ASC precursors enriched for SLE-associated autoreactivity (23, 41, 85).

While containing a relatively small fraction of IgM+ cells, DN cells are largely IgG+ or IgA+ and display a significant degree of somatic hypermutation albeit at a lower level than CD27+ memory cells. Although direct assignments are complicated by the omission of IgD staining in many studies, DN cells are also commonly expanded in patients with chronic HIV viremia and malaria infection (44, 108, 138, 139).

Overall, DN cells in different conditions are enriched in cells with an extended phenotype that includes: CD19hi; CD21lo; CD38+/–; CD24+/–; CD23–; FcRL5+; CD11c++; T-bet++. However, a notable difference between DN cells in SLE relative to HIV infection resides in the absence of FcRL4 in SLE (23, 41) (Figure 2E). The inhibitory FcRL4 receptor is a key phenotypic and functional feature of tissue-based memory cells, a population best defined in mucosal tissues that represented the first example of so-called atypical memory cells (26, 45). From a functional standpoint, the ability of FcRL4 to dampen B cell receptor signaling may account for the inhibited or exhausted function of DN cells reported in chronic HIV infection (93). Their ultimate functional properties however, are likely to be complex and context-dependent as both in these infections and in SLE, DN cells are characterized by high expression of multiple inhibitory receptors including CD32b, CD72, CD22, and PD-1 (138) and at least in HIV, of the inhibitory CD85j (132). Yet, despite the expression of FcRL4, CD85jhi DN cells appear to be activated and comprise the majority of the anti-gp140-specific responses in the early phases (<3 months) of acute HIV infection. However, their relationship with the also CD21lo DN cells previously considered an unproductive repository in chronic HIV infection remains to be clarified (44, 108). Further suggesting that atypical memory cells can be functionally productive in certain settings, FcRL5+ cells with atypical memory markers can be generated through immunization and generate strong recall responses in malaria infection (140). Recent work indicates that the activation of DN cells in SLE may result not only from absence of FcRL4 but also from a more generalized defect in the function of inhibitory receptors and overall hyperresponsiveness to TLR stimulation (41).

Additional heterogeneity within the DN population has been recently established in SLE, where these cells comprise two major subsets on the basis of expression of CXCR5, CD21, and CD11c (41, 95) (Figure 3A). Thus, DN1 cells, representing the large majority of DN cells in healthy subjects and quiescent SLE, express a CXCR5+ CD21+ CD11c– phenotype. In contrast, DN2 cells representing the majority of expanded DN cells in active SLE, display a CXCR5– CD21– CD11c++ phenotype. Notably, DN2 cells also express FcRL5 and this marker can substitute for CD11c in their phenotypic characterization within the appropriate context of other markers. Immunologic, repertoire, and transcriptional characterization of DN1 and DN2 cells suggest that the former subset may represent early activated memory cells (41). Whereas, DN2 cells would represent a primed ASC precursor derived from newly activated naïve cells (which share essentially the same phenotype, with the exception of IgD expression), through an extra-follicular differentiation pathway; a fate consistent with that of CD11c+ extrafollicular plasmablasts in T-independent responses (41, 85, 141).

Currently, atypical memory and tissue-based memory nomenclature appears to be used in the literature interchangeably. Based on the evolving understanding of the different but overlapping populations comprised under these labels, we recommend the use of DN to denote a canonical population that is distinct from conventional naïve and memory cells on the basis of the defining core markers IgD and CD27. We would further advise the recognition of DN1 and DN2 cells based on the relative expression of CD21 or CXCR5 (which largely overlap) and CD11c or FcRL5. We also posit that the atypical memory definition may be unnecessary and possibly misleading, at least for patients with autoimmune diseases, as in such diseases these populations are largely comprised of non-memory cells but rather of activated DN2 effector cells generated through extrafollicular activation. Moreover, these populations are not atypical, but rather part of normal immune responses. We finally recommend that, if so desired, and as indicated by the specific condition under study, the terms atypical memory and/or tissue-based memory be reserved for DN cells with a FcRL4+ phenotype. Finally, we advise against classifying these populations on the basis of a CD27– CD21– phenotype in the absence of IgD staining as such cells would also include IgD+ activated naïve cells. Given the functional uncertainty and implications in different immunological conditions we would argue against attaching additional functional properties, including “exhaustion” or “inhibited,” on the basis of these surface phenotypes alone.

### The Conundrum of Human ABCs and Other Non-discreet Subsets Including T-bet+ and CD21low B Cells

Over the last few years, an interesting population of T-bet+ CD11c+ B cells has gained prominence in mice and by extension, in humans (61, 142–145). Initially described as Age-Associated B cells (ABC) on the basis of their prominence in aging mice, ABC expansions were then identified in younger animals in different autoimmune mouse models and demonstrated to be critical for viral clearance and autoimmune disease. Mouse ABC are TLR-7-driven and differentiate in response to IFNγ and IL-21 stimulation, a differentiation fate that is counteracted by IL-4 (140, 146, 147). More recently, similar populations (including the DN2 cells previously discussed), have been reported in humans and often lumped together as ABC despite growing evidence for the presence of a high degree of heterogeneity within these populations (95, 111, 132, 142). Thus, ABC and “ABC-like” phenotypes have been ascribed, often owing to the expression of one or more ABC markers (preferentially CD21, CD11c, or T-bet), to CD27+ memory cells with a number of studies proposing that human ABCs are predominantly of a CD27+ memory phenotype (108, 109, 111, 148). However, ABC-like populations have also been reported within atypical, tissue-based memory cells (132, 138). This confusion stems largely from the use of different markers to define ABC and in particular, from an over-emphasis on the significance of CD21 downregulation as an indicator of distinct B cell subsets beyond their activation status (Table 1). Indeed, the existing evidence supports the notion that both T-bet and CD11c upregulation as well as, and often times concurrently, CD21 downregulation, does occur in multiple B cell subsets under some activation conditions and does not by itself identify a specific B cell lineage or distinct population (Figure 3). In addition, the concentration of ABC within a given compartment may depend on the pre-determined study in some cases of only some B cell compartments and of the specific subjects and clinical situations studied. For example, it stands to reason that studies of memory recall responses would identify ABC-like cells predominantly within the responding memory cells that expand after immunization (109). Our own results provide some insight into the pattern of expression of T-bet and other associated ABC markers including CD11c, CD21, and FcRL5. In particular, studies in SLE, a condition that uniquely combines strong, simultaneous activation of both new naïve B cells and pre-existing memory cells, indicates that the majority of T-bet++, CD11c++, CXCR5–, CD21lo cells (all ABC-associated markers) reside within activated IgD+ naïve and IgD/CD27– DN2 cells with a smaller fraction of CD21lo cells with intermediate levels of expression of T-bet and CD11c, identified within the CD27+ memory population and in particular within the CD21lo activated memory subset (Figure 3B) (41, 42). Notably, *in vitro* stimulation of naïve, DN cells, and memory cells under conditions known to induce mouse ABC (TLR7, IFNγ, and IL-21), strongly induce a CD19++, CD21lo, CD11c++, T-bet++, FcRL5+ phenotype that closely parallels the *in vivo* phenotype of activated naïve and DN2 cells (41, 95, 140) (Figures 3C–E).

In all, we postulate that the limited use of either CD21, T-bet or CD11c expression is inadequate to identify ABC or other distinct human B cell populations and that the present ABC assignment non-specifically integrates multiple B cell populations. This contention is further supported by recent evidence indicating that multilineage effector B cells can derive from T-bet+ memory precursors (149). A unifying view of the available evidence in humans supports the concept that ABC-like cells represent activated effector B cells induced by TLR7 and driven by IL-21 and IFNγ produced by T_FH_ cells in Th1-type responses within multiple and possibly, all B cell populations (41, 61, 95, 146, 147, 150). Whether the expression of the defining T-bet program or other transcriptional programs determines a distinct lineage or B cell differentiation fate beyond B cell activation and IgG1/IgG3 class switch remains to be determined (146, 151, 152). Given that the expansion of these cells appears to be age-independent in humans (41), we believe that there is no strong rationale nor biological basis for this designation in human B cells.

### Antibody-Secreting Cells (ASC: Canonical Phenotype: CD19+/–, IgD–, CD27++, CD38++, CD24–)

**Figure 4:**
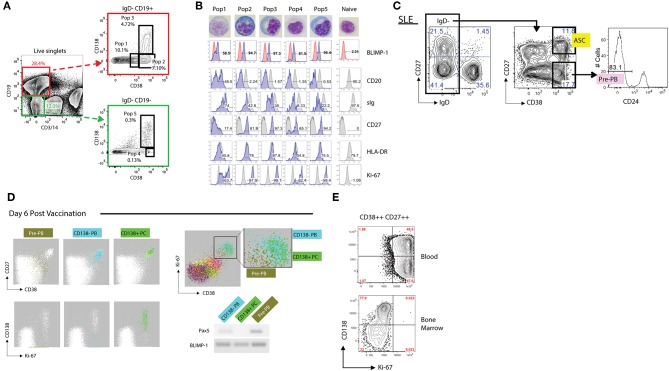
Phenotypic analysis of antibody secreting cells (ASCs). **(A)** Representative FACS gating strategy (from an SLE patient) for discerning both CD19+ and CD19– ASCs populations from the blood. Utilizing CD3 and CD14 as “dump” channel markers CD19+ and CD19– (non-T cells, non-monocytes) B cell populations can be extracted from the data. Subgating on the IgD- fraction and utilizing CD138 vs. CD38 allows for the breakdown of ASC populations into five fractions: Pop1, CD19+ CD38mid CD138–; Pop2, CD19+ CD38hi CD138–; Pop3, CD19+ CD38hi CD138+; Pop4, CD19– CD38hi CD138–; Pop5, CD19– CD38hi CD138+. **(B)** Morphological and further phenotypic alterations in circulating ASC populations in **(A)** as compared to circulating naïve B cells, revealing plasmablast/plasma cell programming with high BLIMP-1, CD20lo/–, sIglo, and CD27++ expression, with the association of HLA-DR and Ki-67 positivity. **(C)** Gating strategy of an SLE patient revealing a sizeable presence of CD19+ IgD– CD27– CD38hi CD24– pre-PB B cells. **(D)** FLOCK analysis of a healthy donor 6 days post vaccination exhibiting CD38mid pre-PB, CD138– plasmablasts (PB), and CD138+ plasma cells (PC) on a CD27 vs. CD38 dot plot. In circulating ASCs Ki-67 was representative of both CD138– PB and CD138+ PC, whereas pre-PB are Ki-67+ without the CD138 expression. Pre-PB show an enrichment for BLIMP-1 expression while simultaneously expressing Pax5, signifying a transitional state toward an ASC fate. **(E)** CD138 vs. Ki-67 plot in subgated circulating and bone marrow ASCs representing the differences in proliferation status, with bone marrow ASCs exhibiting little Ki-67 expression. This dichotomy likely signifies newly generated vs. resident PC populations.

Antibody-Secreting cells (ASC), encompass both proliferative cells (plasmablasts; PB), at different stages of differentiation from either naïve or memory cells as well as resting, mature plasma cells (PC). The ultimate classification of ASC as either PB or PC varies in the literature with some authors basing this adjudication on proliferative status (typically on the basis of Ki-67 expression), while others rely on the expression of CD138 to enumerate mature PC. BLIMP-1, IRF4, and XBP1 are the traditional transcription factors important for ASC differentiation (153–160). While it seems clear that CD138 expression identifies more mature PC and is globally expressed by bone marrow-long lived PC, it has also become evident that CD138 is expressed by a fraction of circulating, proliferative PC both in response to immunization as well as in active SLE (85, 161–164) (Figures 4A–D). Moreover, CD138 can be induced by *in vitro* stimulation of blood memory cells, thereby indicating that the expression of this marker is not restricted to fully differentiated, mature, resting PC (162, 164–166). As shown in Figures 4B,E, during active immune responses, including active SLE, proliferative CD138+ cells can account for a large fraction of all circulating ASC. By and large, the expression of Ki-67 overlaps with the expression of HLA-DR antigens and the latter therefore represents a useful surrogate for recently formed proliferative PB (161), obviating the need for intracellular staining. For the purpose of this review, we will focus on peripheral blood ASC subsets.

All peripheral human ASC (including those in lymphoid tissues and bone marrow), share high expression levels of CD38 and accordingly, this marker is critical for their identification (163, 167–170). Other human CD19+ B cells with high expression levels of CD38 include transitional cells which express IgD and CD24 and germinal center cells, which are usually absent in the blood. Although IgD+ ASC have been previously described, only a small fraction express BLIMP-1 and are rarely found in the periphery (131, 171, 172). Hence, all circulating ASC can be identified using the core markers under a CD19+, IgD–, CD38++ phenotype (Figure 4A). Human ASC also express high levels of CD27 and down-regulate CD20 expression. Of note, core markers also identify an additional population of proliferative IgD– CD38+/++ CD24– cells expressing low levels of CD27 (Figure 4A). Initially thought to be restricted to the human tonsil (169), circulating pre-PB have also been identified using automated multidimensional analysis of human vaccine responses where it behaves with a kinetics similar to conventional PB responses (163). These cells upregulate BLIMP-1 expression while maintaining expression of the B cell transcription factor Pax5 and are therefore, likely to represent PB precursors (pre-PB) (162). Of note, a significant fraction of pre-PB (up to 40%; Figures 4C,D), maintain expression of CD20 and accordingly, would be missed using a CD20– gate (162).

Finally, recent work has clearly identified populations of ASC lacking CD19 expression, a feature known to define terminally differentiated bone marrow plasma cells including multiple myeloma PC (173). However, in healthy subjects, CD19– PC are heterogenous and contain both CD138– and CD138+ cells with the latter fraction representing the source of human long-lived PC (40). CD19– PC can also be identified in the human blood in response to immunization and can be generated in culture (162, 164, 166).

### B Regulatory Cells (Breg; No Specific Canonical Phenotype)

B regulatory cells (Breg), can be best defined as cells with the ability to inhibit pro-inflammatory monocytes and T cell responses although multiple other cellular targets have also been proposed, including plasmacytoid dendritic cells and anti-tumor cytotoxic T cells (48, 174). Following early descriptions for a central role of IL-10-producing Breg cells in the suppression of autoimmune diseases (175), multiple other cytokines including TGFβ and IL-35 as well as other mediators have been reported (48, 176). In addition Bregs can also suppress autoimmunity through the modulation of regulatory iNKT cells through CD1d-mediated lipid presentation (177). Intriguingly, CD1d Bregs were found in one study to be deficient in SLE and their expansion post-BCDT to correlate with favorable clinical response to rituximab (177). While CD1d+ Bregs concentrate within the CD24++ CD38++ transitional B cells proposed to represent a major IL-10-producing Breg human population (178), this study did not address the production of this cytokine.

From a phenotypic standpoint, Breg function has been identified within multiple human B cell populations including: CD38++C24++ transitional cells, naïve B cells, CD27+CD24high memory cells including B10 cells, CD27+ CD1d+ (marginal zone-like) memory cells, CD27+ CD5high PD-1high memory cells, and a TIM-1+ population with heterogeneous expression of CD27, CD24, CD38, CD1d and CD5 (48, 174, 178–186). Yet, even within those populations, Bregs may account for <20% of B cells and thus, overall, they represent a small fraction of human B cells (48, 184). Currently, no set of surface phenotypic markers can identify Bregs and we agree therefore, with the expert recommendation that an accurate enumeration of Bregs, short of the ideal functional characterization, should rest on measurements of cytokine production at the single cell level by intracellular staining of regulatory cytokines such as IL-10 and IL-35 (48, 184). Substantial experimental evidence indicates that such measurements require *in vitro* short-term stimulation to reveal either cells containing pre-formed cytokines or longer stimulation to identify Breg precursors (B10 and pre-B10, respectively, for IL-10-producing Breg) (181, 184).

Finally, a number of studies have identified regulatory PB and PC whose function is mediated through either IL-10 or IL-35 and whose phenotype may include expression of PD-L1 and IgA (187–190). Regulatory PC appear to play an important role in autoimmune diseases and cancer immune responses where they may promote tumor progression (191).

## Concluding Remarks

As our understanding of human B cell biology expands and new novel populations are discovered, it is critical to not lose sight of the need for phenotypic standardization. This all-important feature to experimental design will not only allow for the inter-laboratory interpretability but place the field of B cell immunology in a position to bound forward with limited hindrance on progress. Within this review we have offered a suggestion of standardizing B cell phenotyping with a core stain of seven surface markers, and also given strong support for the expandability of subgating beyond these core markers in a variety of B cell populations. Additionally, we raise the need for awareness that not all current surface antigens being utilized for B cell subgating strategies are likely sufficient to conclusively prove the existence of independent functional populations. Taken together we hope that this review may serve as a reference for future experimental designs and a springboard for phenotypic B cell normalization.

## Ethics Statement

These studies were conducted from fully consented patients under ethical and safe protocols in accordance with the Declaration of Helsinki, and with the approval of the Institutional Review Boards of Emory University and the University of Rochester.

## Author Contributions

IS, CW, SJ, KC, and FL contributed to the literature review and manuscript preparation. IS, SJ, CW, CT, JH, KC, and MW were responsible for figure design and legend assembly.

### Conflict of Interest

The authors declare that the research was conducted in the absence of any commercial or financial relationships that could be construed as a potential conflict of interest.
